# Increased flux pinning force and critical current density in MgB_2_ by nano-La_2_O_3_ doping

**DOI:** 10.1088/1757-899x/756/1/012019

**Published:** 2020-07

**Authors:** Danlu Zhang, Fang Wan, Michael D. Sumption, Edward W. Collings, CJ Thong, Matt Rindfleisch, Mike Tomsic

**Affiliations:** 1 CSMM, Materials Science and Engineering, The Ohio State University, Columbus, OH 43220, USA.; 2 Hyper Tech Research Inc., Columbus, OH 43228, USA.

## Abstract

MgB_2_ superconducting wires and bulks with nano-La_2_O_3_ addition have been studied. A series of MgB_2_ superconducting bulk samples with nano-La_2_O_3_ addition levels of 0, 5, 7, 18wt% were prepared. AC resistivity data showed slight increases of *Bc*_*2*_ and unchanged *B*_*irr*_ for the bulk samples with doping levels lower than 7 wt% and decreased critical fields for the heavily doped (18 wt%) bulk. X-ray diffraction (XRD) showed the presence of LaB_6_ in the nano-La_2_O_3_ doped MgB_2_ bulk samples and decreased MgB_2_ grain size in nano-La_2_O_3_ doped bulks. Monocore powder-in-tube (PIT) MgB_2_ wires without and with 5 wt% nano-La_2_O_3_ addition (P-05) were prepared for transport property measurement. 2mol%C-doped *Specialty Materials Inc.* (SMI) boron powder was used for wire P-05 and previously prepared control wires (control wires were made without the addition of nano-La_2_O_3_ powder, W-00 and P2). Low field magnetic properties were obtained from magnetization loop (M–H), transport critical current density (*J*_*c*_) was measured at 4.2 K for the nano-La_2_O_3_ doped PIT wire (P-05) and the control samples (P2 and W-00). The transport critical current density *J*_*c*_ (B) of P-05 at 4.2 K and 8 T (6.0 ×10^4^ A/cm^2^) was twice that of the control wire. The critical magnetic fields (*Bc*_*2*_ and *B*_*irr*_) of P-05 and the control sample P2 were compared. The critical fields of P-05 were slightly less than those of P2. Kramer-Dew-Hughes plots indicated a change from surface pinning to a mixture of volume pinning and surface pinning. It is shown that enhancement of P-05’s transport properties is due to additional flux pinning by the fine-size rare-earth borides rather than enhanced *Bc*_*2*_ or *B*_*irr*_.

## 1. Introduction

Since the discovery of MgB_2_ superconductors in 2001 [1], substantial improvement on the material has been achieved in terms of critical field, transport property, wire manufacture processes. Due to the relatively high *T*_*c*_ (39 K) in MgB_2_ and the shortage of liquid helium worldwide, MgB_2_ is particularly useful for helium-free MgB_2_ MRI magnets [2] and is expected to replace Nb-based MRI magnets in the future. To reach these goals, improvements in the critical current density (*J*_*c*_) of MgB_2_ conductors are necessary.

Numerous approaches have been taken to produce MgB_2_ materials with high transport properties: (1) chemical doping of MgB_2_ wires [3–5]; (2) cold pressing [6–7]; (3) hot isostatic pressing (HIP) [8–9]; (4) the introduction of the internal magnesium diffusion (IMD) method to address porosity and connectivity issues [10–12]. So far, the best “non-barrier” transport *J*_*c*_ values were obtained by the advanced internal magnesium infiltration (AIMI) approach, with the addition of C and Dy_2_O_3_ (1.07 × 10^5^ A/cm^2^) at 10 T, 4.2 K, [13]. The AIMI technique has the benefit of forming dense MgB_2_ layers with improved longitudinal connectivity compared to the extrinsic conventional powder-in-tube (PIT) method, which usually produces wires with randomly connected MgB_2_ fibers. On the other hand, the PIT method is favored as a simple and inexpensive approach to studying wire properties in response to chemical doping.

Many chemicals have been added to MgB_2_ in the past 18 years to study their effects on the resultant transport and other superconducting properties, such as upper critical field (*Bc*_*2*_) [14–15], and irreversibility field (*B*_*irr*_) [16–17]. Generally, improvements in *J*_*c*_ have been attributed to the improvements of either *Bc*_*2*_ or *B*_*irr*_. Till now, C has been shown to be the most effective doping element for the enhancement of *Bc*_*2*_ [18] in MgB_2_ materials. Many MgB_2_ wires with high transport properties were doped with C or C-containing materials. In addition to C and C containing materials, doping with Dy_2_O_3_ has shown to increase both *J*_*c*_ and *B*_*irr*_ [19–20]. In particular, Chen *et al.* [19] and Li *et al.*[20] both demonstrated the effectiveness of a combination of flux pinning by Dy_2_O_3_ and carrier scattering by C. The effects on the transport *J*_*c*_ of rare earth oxide additions such as Pr_6_O_11_ [21–22], CeO_2_ [23], Eu_2_O_3_ [24] have also been studied.

This paper describes the effect of adding nano-La_2_O_3_ dopants on superconducting and structural properties in MgB_2_ superconductors. La_2_O_3_ was chosen because some rare-earth oxide additions have improved *J*_*c*_ and *Bc*_*2*_/*B*_*irr*_ [19–24] in MgB_2_ superconductors. Besides, La_2_O_3_ doping has been previously studied for MgB_2_ tapes and nano LaB_6_ flux pinning centers as well as increase of *J*_*c*_ in the doped tapes were observed. Therefore, the effects of La_2_O_3_ doping in MgB_2_ wires and bulks were studied here to fully explore the doping effect as well as the optimum doping level. Consequently, four different doping levels (0, 5, 7,18 wt%) were chosen for the bulks and 5 wt% was chosen to make PIT wires based on the property measured for bulks with the same doping level. Transport *J*_*c*_ in the 5 wt% nano-La_2_O_3_ doped monocore PIT-processed MgB_2_ wire increased to twice the value of the control sample at 4.2 K, 8 T.

## 2. Experimental

### 2.1. Sample preparation

#### 2.1.1. Bulk samples.

Four bulk samples B-00 (0 wt% La_2_O_3_ added), B-05 (5 wt% La_2_O_3_ added), B-07 (7 wt% La_2_O_3_ added) and B-18 (18 wt% La_2_O_3_ added) were made. The precursor powders used were 2 mol% C doped SMI boron powder, Mg powder (325 mesh) from Alfa Aesar, and nano-level (10–100 nm) La_2_O_3_ powder manufactured by US Research Nanomaterials Inc. The mole ratio of Mg to B powder used was 1: 2. The powder was mixed inside a glove box, and then transferred to a hydraulic press for densification at 5000 psi (or 34.5 MPa). The resulting pellets were heat treated at 850 °C for 30 minutes in flowing Ar, followed by furnace cooling.

#### 2.1.2. Wire Samples.

The wire samples were prepared by Hyper Tech Research using their well-known “continuous tube filling and forming” (CFTT) powder-in-tube (PIT) process [25] followed by wire drawing to 0.83 mm in diameter. Selected for controls were two samples from previous studies. Designated P2 [26] and W-00 [27] precursor powders were 2 mol% C doped B from SMI and Mg powder as in the undoped bulk. The sample specifications are listed in Table 1.

### 2.2. Measurements

#### 2.2.1. X-Ray diffraction (XRD) measurement.

The powdered bulk samples were scanned on a Rigaku Miniflex 600 XRD machine at a scan rate of 5 deg / min. Phases and peaks were studied with the help of PDXL software.

#### 2.2.2. AC resistivity.

The AC resistivity vs temperature measurements on the bulk samples were performed on a Quantum Design Model 6000 Physical Property Measuring System (PPMS). The bulk samples were cut into rectangular prisms and four-point probe measurements were used to obtain sample resistance of 5 K to 45 K. The usual “10% normal resistivity” and “90% normal resistivity” rules were used to obtain *B*_*irr*_ and *Bc*_*2*_, respectively.

#### 2.2.3. Transport J_c_ measurement.

The transport *J*_*c*_ measurements were carried out in transverse magnetic fields of up to 12 T in a liquid He bath at 4.2 K on samples 50 mm long with a gauge length of 5 mm and an electric field criterion of 1 μV/cm. Measurements were also done using a gauge length of 4 mm. The layer *J*_*c*_ of the CTFF PIT strands were calculated using the critical transport currents divided by the MgB_2_ layer area.

#### 2.2.4. Magnetic J_c_ measurement.

Magnetization-Magnetic field (M-H) loops were measured under VSM mode in a Quantum Design Model 6000 Physical Property Measuring System (PPMS) on sample P-05 3.2 mm long. Magnetic *J*_*cm*_ at 4.2 K was extracted from M-H loop at a ramp rate of 10 mT/s in transverse fields of up to 9 T. Based on Bean’s model [28], the 4.2 K magnetic *J*_*cm*_ for the superconducting CTFF monocore strand and was derived from:

$$J_{cm}=\frac{3\cdot \pi \cdot \Delta M}{4\cdot d},$$

where d is the diameter of the MgB_2_ layer inside the wire and Δ*M* is the full height of the M-H loop at a certain field. The magnetic *J*_*cm*_ played a role in the pinning force calculation (see section 3.4).

## 3. Results and Discussion

### 3.1. Bulk sample’s magnetic and structure properties

**Figure 1.**
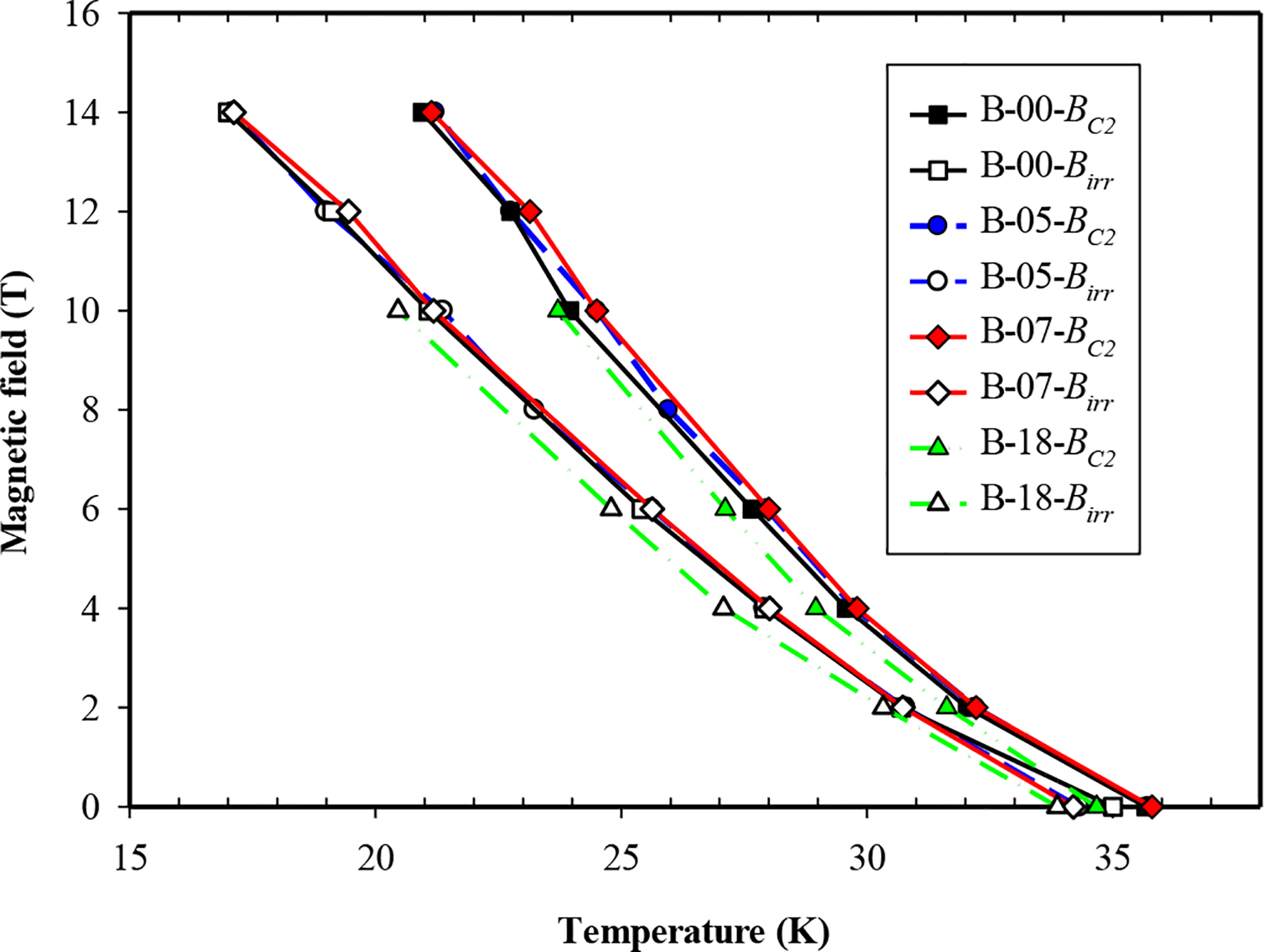
Critical magnetic fields as a function of temperatures for undoped bulk sample (B-00) and doped samples (B-05, B-07, B-18).

*Bc*_*2*_ and *B*_*irr*_ values were obtained for MgB_2_ bulks doped with nano-La_2_O_3_ and the control bulk sample (B-00) at temperatures of 16 K ~ 36 K, Figure 1. *B*_*irr*_ stayed unchanged for B-05 and B-07. *Bc*_*2*_ increased by 0.2 T with the addition of 5 wt% La_2_O_3_ and increased by 0.4 T with addition of 7 wt% La_2_O_3_. However, both *Bc*_*2*_ and *B*_*irr*_ decreased significantly in response to doping with 18 wt% La_2_O_3_. Based on the data in Figure 1, it can be seen that *T*_*c*_ of the doped bulks did not change compared to the control bulk sample. This is similar to the unchanged *T*_*c*_ observation on Dy_2_O_3_ doped MgB_2_ bulks [29], meaning no significant atomic substitution occurred on MgB_2_ host lattice sites.

**Figure 2.**
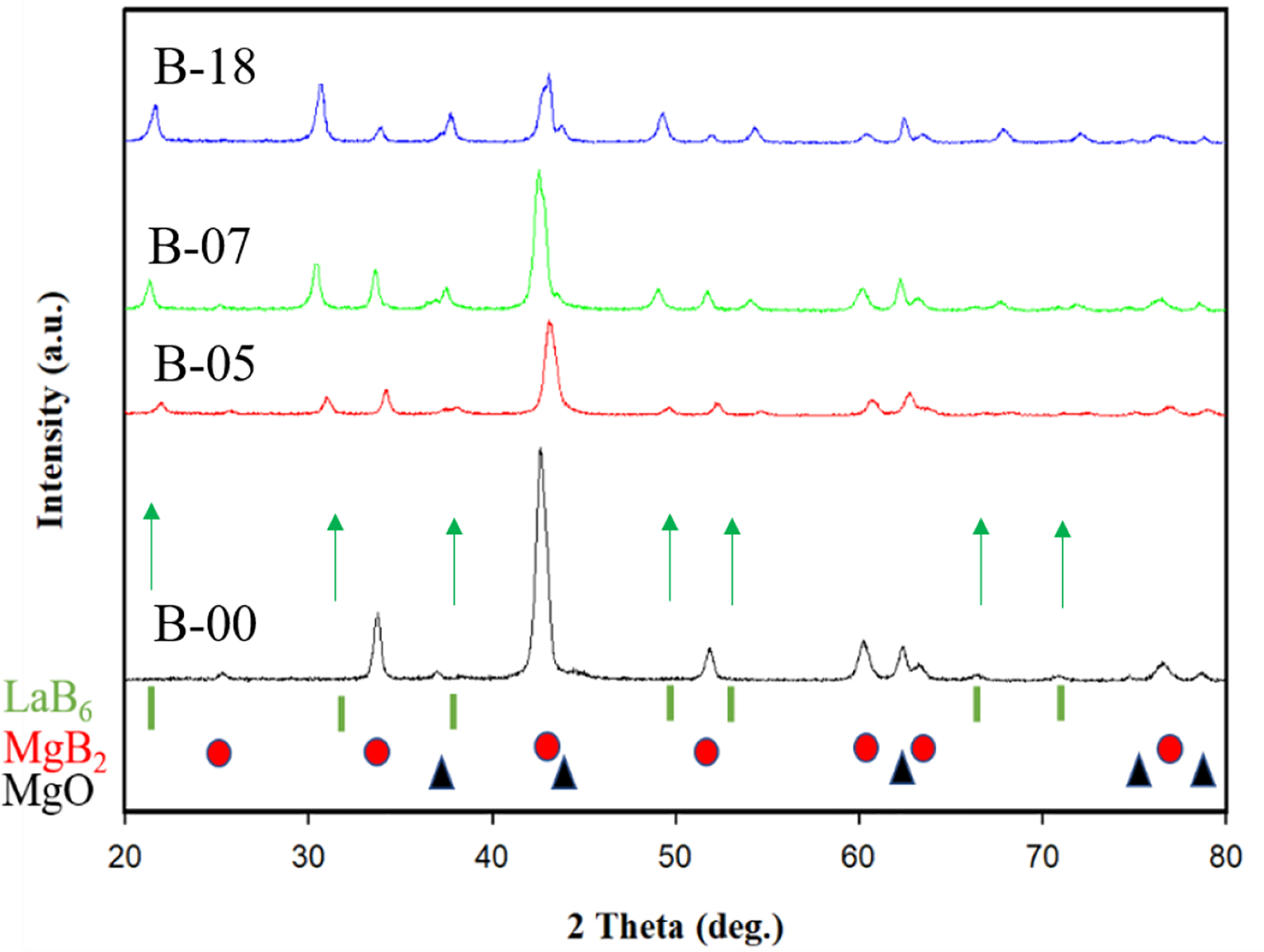
XRD patterns for undoped MgB_2_ bulk and MgB_2_ bulks doped with nano-La_2_O_3_.

**Figure 3.**
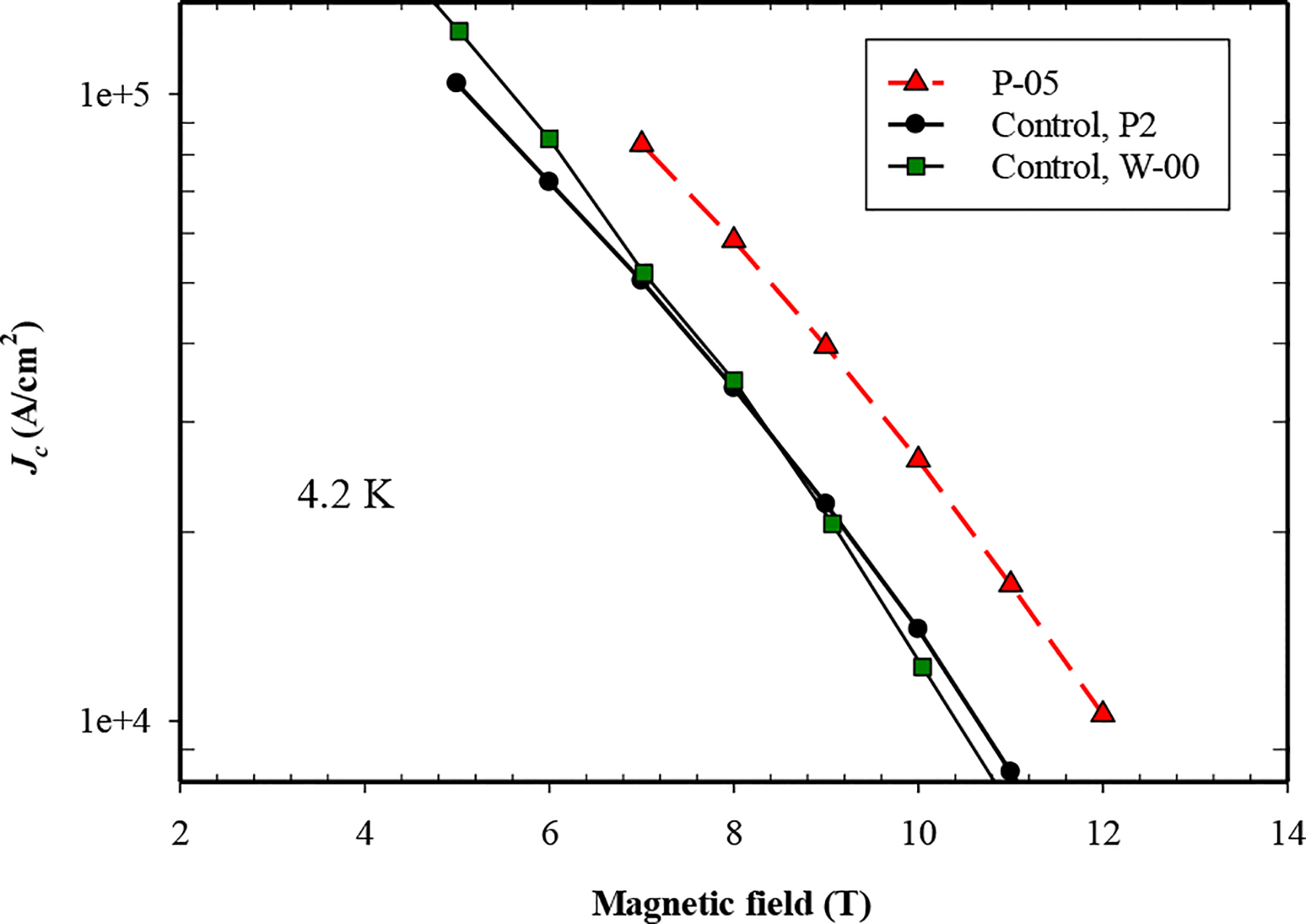
Critical current density (*J*_*c*_) as a function of magnetic field for control wire and the La_2_O_3_ doped sample P-05 a 4.2 K.

Phases and peaks in the MgB_2_ bulk samples have been studied with the aid of XRD. The patterns shown in Figure 2 indicates the presence of LaB_6_ in all doped bulk samples (shown by green arrows), presumably there are pinning centers. The MgB_2_ peaks of B-05 and B-07, which is consistent with the minor increases of *Bc*_*2*_ seen in Figure 1. The lattice shifts are smaller than those observed by Gao *et al.* [30] in MgB_2_ tapes with acetone and La_2_O_3_ additions. Usually MgB_2_ peak shifts observed in XRD patterns are caused by atomic substitution and/or strain. The shifts seen in Figure 3 is probably due to the lattice strain generated by the nanoparticles (such as LaB_6_) since *T*_*c*_ was shown to be unchanged in doped samples. MgB_2_ grain size was estimated based on XRD data using William-Hall method, Table 1. As the La_2_O_3_ doping level increases from 0 wt% to 7 wt%, MgB_2_ grain size decreased from 20.6 nm to 7.6 nm. The grain size of MgB_2_ was 17 nm in the heavily doped sample. Yuan [27] observed same trend in his Dy_2_O_3_ doped MgB_2_ bulks [27]. He attributed this grain size reduction to secondary phase DyB_6_ nanoparticles. Here, the reduction of grain size in La_2_O_3_ doped MgB_2_ bulks might be due to the secondary phase LaB_6_, which inhibits the grain growth and pins fluxons.

### 3.2. Wire strength, transport and magnetic properties

This paper also describes the effect of 5 wt% nano-La_2_O_3_ addition to MgB_2_ PIT wires. Two previously-made control samples (P2 [26] and W-00 [27]) were used for reference. These two wires were interchangeably used not only because the compositions are the same and the heat treatment procedures are very similar, but also because measured data from two controls combined can offer complete data for comparison purpose in this paper. Though they were manufactured from different batches, these mono PIT wires (with the use of 2mol%C-doped B powder) usually possess consistent properties. The transport properties of PIT MgB_2_ wire P-05 and undoped wires P2 and W-00 were measured at 4.2 K in transverse magnetic fields up to 12 T, Figure 3. It can be seen that La_2_O_3_ doping resulted in a significant increase in *J*_*c*_. In particular, at 8 T and 4.2 K, the *J*_*c*_ of P-05 is 6.0 ×10^4^ A/cm^2^, about twice that of the control wire P2. It is important to keep in mind that AIMI approach usually produces MgB_2_ wires with higher *J*_*c*_ compared to conventional PIT method for wires with same composition and heat treatment procedure because of the enhanced connectivity. Thus, AIMI-produced wires are not discussed here.

### 3.3. Wire sample: Critical fields and flux pinning

**Figure 4.**
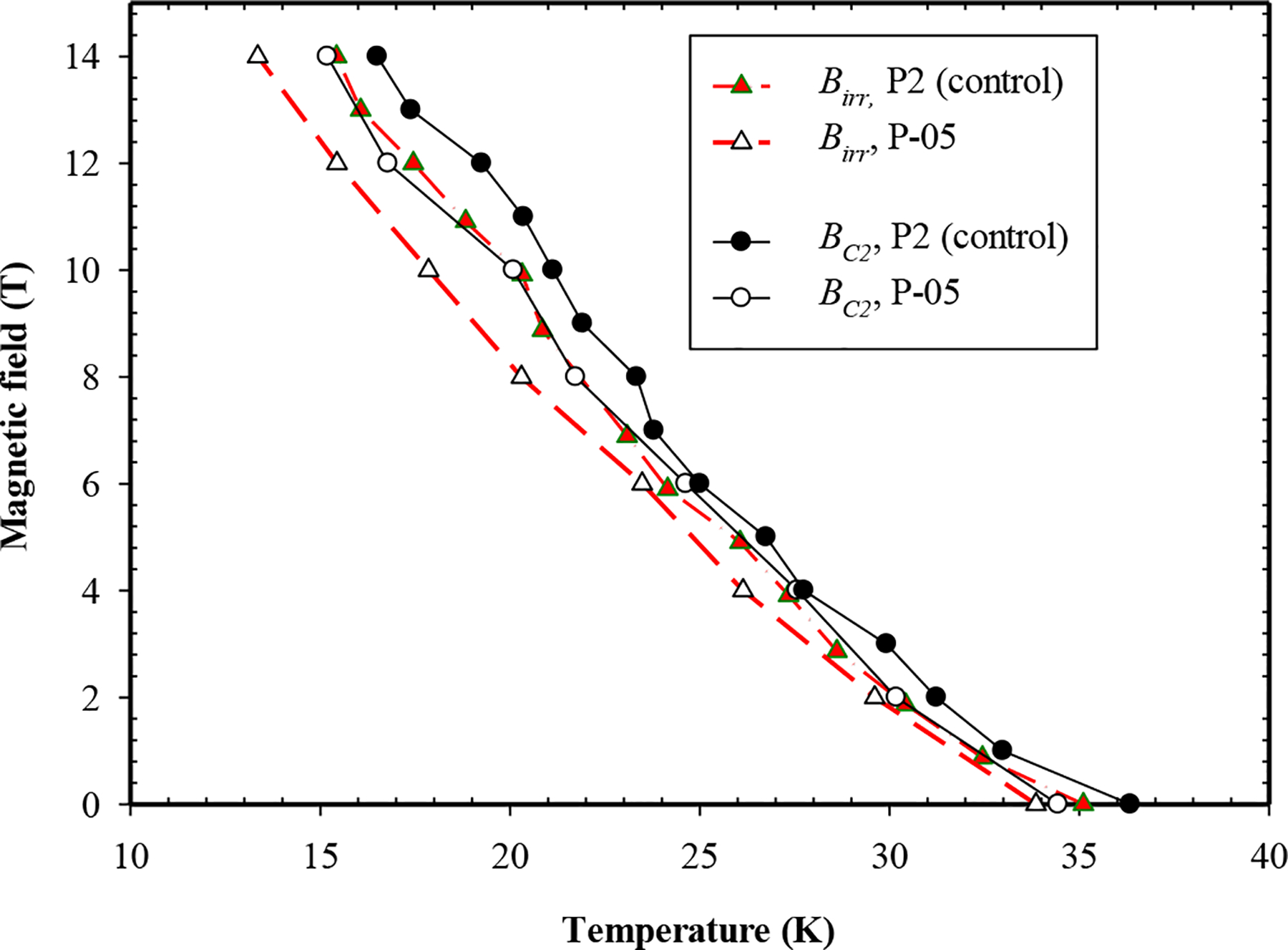
Upper critical field and irreversibility field for the control sample and nano-La_2_O_3_ added PIT MgB_2_ wire P-05.

**Figure 5.**
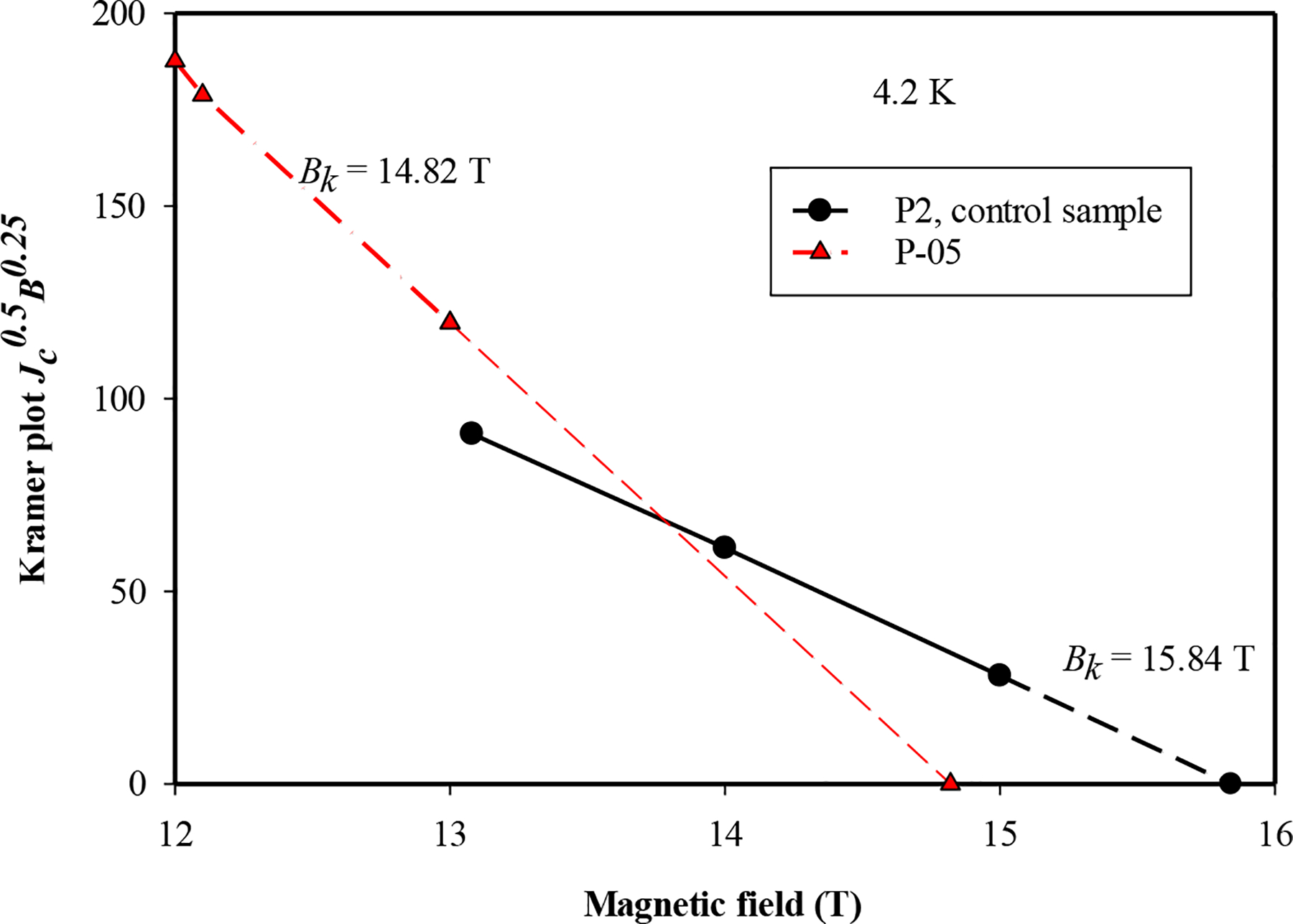
Kramer plot (*J*_*c*_^0.5^B^0.25^ vs. B) for control wire sample and P-05 at 4.2 K. The irreversibility fields based on the Kramer model [31], *B*_*k*_, were taken as the extrapolated x-axis intercepts of linear fittings (evenly spaced dashed lines).

In order to enquire into the factors responsible for the enhancement of *J*_*c*_, the critical fields (*Bc*_*2*_ and *B*_*irr*_) were measured via resistivity approach in the PPMS in Figure 4. The *T*_*c*_ of P-05 decreased with the addition of La_2_O_3_ and its *Bc*_*2*_ decreased by about 1 T at lower temperatures and very insignificantly at higher temperatures. Likewise, its *B*_*irr*_ decreased by around 0.6 T at lower temperatures and by a smaller amount at higher temperatures. A decrease in the Kramer field, *B*_*k*_ (a surrogate for Birr) of 1.0 T at 4.2 K (Figure 5) confirms the above result. The relatively larger Bc_2_ values in the undoped wires could be due to the existence of nano-level electron scattering centers (e.g., grain boundaries, point defects, volume defects and secondary phases). Since these two wires shown on Figure 4 are manufactured at different heat treatment conditions, it is very likely that fine secondary impurity phases such as MgO, B_2_O_3_ were introduced in the undoped wire P2 and therefore enhanced *Bc*_*2*_ to a slight degree.

Two common mechanisms for enhancing *J*_*c*_ in MgB_2_ wires are to enhance *B*_*irr*_ or enhance *Bc*_*2*_. Here, we propose another mechanism for enhanced *J*_*c*_ in MgB_2_ wires with nano-La_2_O_3_ doping. It is well known that enhanced transport *J*_*c*_ by way of C doping in MgB_2_ wire can be attributed to enhanced *B*_*c2*_ by way of enhanced scattering effect [16, 19]. The increased transport *J*_*c*_ in Dy_2_O_3_-doped MgB_2_ wire was attributed to an increase in *B*_*irr*_ [19–20]. With regard to La_2_O_3_ doped wire samples, as mentioned above, the 4.2 K, 8 T, *J*_*c*_ of P-05 was about twice that of the undoped control wire. But this increase is accompanied by no changes or decreases in *B*_*c2*_ and *B*_*irr*_ (1 T and 0.6 T, respectively, at lower temperatures), Figure 4. Clearly, a mechanism other than critical field is responsible for the increase in *J*_*c*_; flux pinning is the obvious choice.

To further study the phenomena, Kramer plot has been made, A Kramer Plot, *J*_*c*_^0.5^B^0.25^ vs. B, was shown in Figure 5. The irreversibility fields based on Kramer model [31], *B*_*k*_, was taken at the x-axis intercepts of linear fittings (evenly spaced dashed lines) on Figure 5. *B*_*k*_ of the doped wire has a lower value than the control wire value, which agrees with the critical field analysis done above on the wire samples.

### 3.4. Flux pinning in response to La_2_O_3_ doping

**Figure 6.**
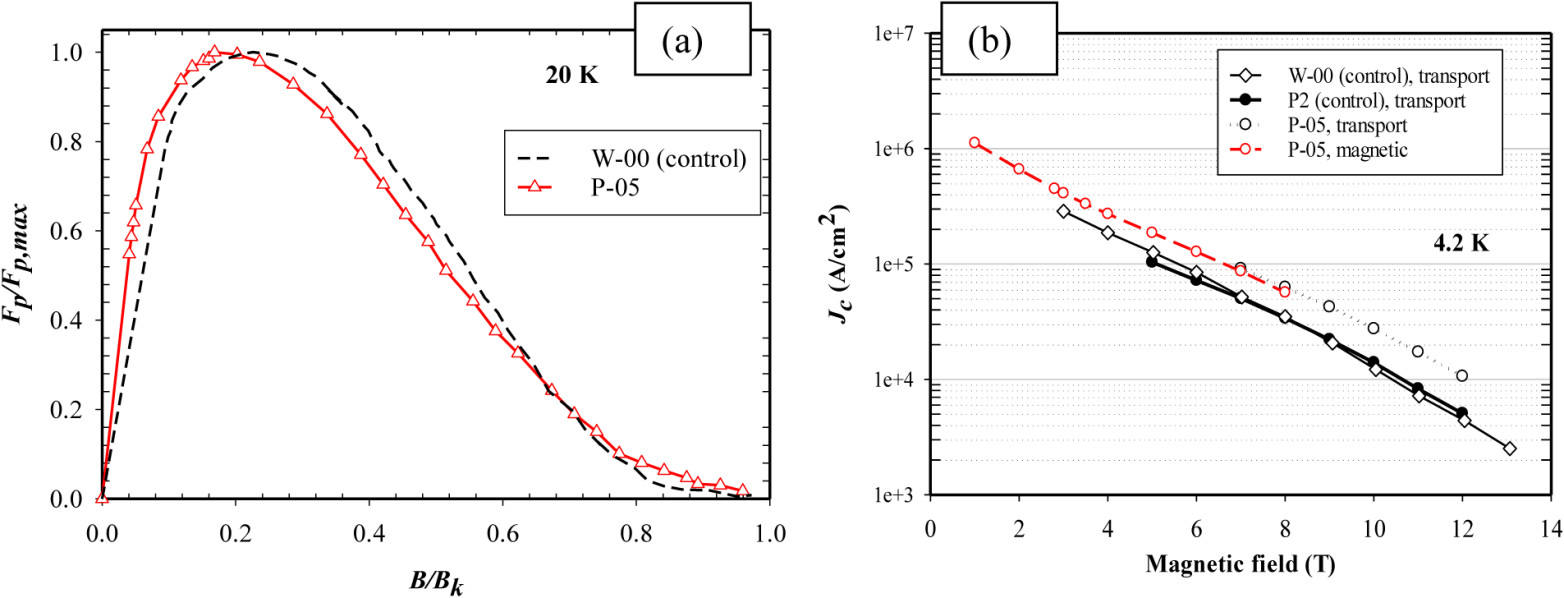
(a, left) Normalized flux pinning force as a function of reduced field for control sample and the doped wire, P05, based on the magnetic data; (b, right) Critical current density measured for wire P-05 using both magnetic measurement and transport measurement.

Using data derived from the M-H loop, the normalized flux pinning force density *F*_*p*_/*F*_*p,max*_ was plotted against normalized magnetic field *b*=*B*/*B*_*k*_ at 20 K to avoid flux jump effect, Figure 6(a). *B*_*k*_ here was derived based on magnetic measurement conducted at 20 K in PPMS. Figure 6(b) showed the *J*_*c*_ data masured by transport and magnetic methods. Apparently, magnetic measured *J*_*c*_ agrees well with the transport data for P-05. Based on Dew-Hughes’s analysis [32] about *f*_*p*_ ∝ *b*^1/2^(1 − *b*)^2^, the curve for the control sample which peaks at *B*/*B*_*k*_= 0.2 is indicative of grain boundary pinning. In contrast, the curve for P-05 wire peaked at around *b* = 0.168. This phenomenon has been observed in Dy_2_O_3_ doped samples [29] and Yang *et al.* [29] stated that two reasons can be responsible for this behaviour: (1) The doped sample might contain a set of local *B*_*k*_*s* instead of one distinct value, which can lead to an artificial error in the estimation of the peak positions; (2) The deviation from *b*_*peak*_ = 0.2 might be due to other pinning mechanisms (e.g., normal volume pinning in which *f*_*p*_ maximizes at b < 0.2 due to anisotropy [33]) in association with the GB pinning. The presence of LaB_6_ peaks in all the XRD patterns of the doped bulk samples strongly suggests that LaB_6_, formed by reaction with the B powder, would also be present in the doped wires and as such would be responsible for the observed volume pinning.

## 4. Concluding Discussion

Four MgB_2_ bulk samples with 0–18 wt% nano-La_2_O_3_ additions and a monocore PIT MgB_2_ wire with 5 wt% nano-La_2_O_3_ addition were prepared for measurements of *J*_*c*_, *B*_*irr*_, *Bc*_*2*_ and magnetic and transport *J*_*c*_ (see Table 1). Resistive measurements of *B*_*irr*_ and *Bc*_*2*_ in response to La_2_O_3_ doping were made in the PPMS on the bulk and wire samples. For the bulks *B*_*irr*_ remained unchanged in B-05 and B-07 but decreased in B-18; *Bc*_*2*_ increased by 0.2 T in B-05 and 0.4 T in B-07 but also decreased in B-18. For the wires (controls and P-05) *B*_*irr*_ decreased by ~0.6 T at low temperatures and a smaller amount at high temperatures. *Bc*_*2*_ decreased by 1 T at low temperatures (resistive results and Kramer plot) and insignificantly at high temperatures. The 4.2 K, 8 T, *J*_*c*_ of P-05 was about twice that of the undoped control. There is no correlation between changes in the critical fields and *J*_*c*_. On the other hand, the occurrence of LaB_6_ in the XRD patterns and the shapes of the normalized flux pinning curves (after Dew-Hughes) indicate that the increase in *J*_*c*_ is a consequence of increased flux pinning in the doped wire.

## Figures and Tables

**Table 1.** Info on bulk and PIT wire samples

Sample Form	Sample Name	Wt% La_2_O_3_	Heat Treatment	Crystallite size (nm)
Bulk	B-00	0	30min/850°C	20.6
Bulk	B-05	5	30min/850°C	19.8
Bulk	B-07	7	30min/850°C	7.6
Bulk	B-18	18	30min/850°C	17
Wire	P2*	0	20min/675°C	-
Wire	W-00*	0	60min/650°C	-
Wire	P-05	5	60min/650°C	-

* Previously made/published [26][27]

Both referred to as “Control”
